# Student Potential for Self-assessment in a Clinical Dentistry Practical Training Course on Communication Skills

**DOI:** 10.1007/s40670-020-01061-5

**Published:** 2020-08-20

**Authors:** Asami Iguchi, Yuh Hasegawa, Kazuyuki Fujii

**Affiliations:** 1 School of Life Dentistry at Niigata, The Nippon Dental University, 1-8, Hamaura-cho, Chuo-ku, Niigata, 951-8580 Japan; 2 The Nippon Dental University College at Niigata, 1-8, Hamaura-cho, Chuo-ku, Niigata, 951-8580 Japan

**Keywords:** Dental students, Self-assessment, Assessment, Communication skills, Dental education

## Abstract

This study aimed to investigate student potential for self-assessment in a clinical dentistry practical training course focused on communication skills. Participants were 124 fourth-year students (70 males, 54 females; all Japanese) in 2017 and 2018 at the Nippon Dental University, School of Life Dentistry at Niigata. Participating students belonged to different cohorts in 2017 and 2018. Participants were asked to complete a self-evaluation sheet at the end of each unit of the course. Their self-evaluation scores and the faculty evaluation scores for each student for Units 1-1, 1-2, and 1-3 were statistically analyzed. The results showed that females tended to rate themselves significantly higher than males. Furthermore, there were significant differences in evaluation scores between students and faculty for nine of 11 evaluation items for male students and 10 of 11 items for female students in Unit 1-3. Faculty expectations increased from Unit 1-1 to Unit 1-3, although students were satisfied with their performance and had a sense of achievement. However, students’ actual performance was below faculty expectations, suggesting faculty evaluations were stricter than students’ self-evaluation. Self-assessment may enhance students’ ability for self-directed learning and may also inform how faculty can effectively educate dental students. Dental educators should support students to increase their levels of self-efficacy, which will enhance their self-evaluation skills.

## Introduction

Many studies around the world have addressed students’ self-evaluation in pharmaceutical education [1–8], medical education [9–14], and dental education [15–21]. Dentistry is a self-analytic profession, and dentists must be able to properly evaluate every procedure performed. Self-assessment is a critical skill that dentists must have to be competent oral healthcare providers [17]. Hadid [10] defined self-assessment based on a report by McMillan and Hearn [22] as being a process by which students monitor and evaluate the quality of their thinking and behavior when learning, and identify strategies that improve their understanding and skills. Dental students’ ability to self-evaluate their work may be an effective learning tool as it enhances their performance in each step [16]. Accurate self-assessment reflects an ability to accurately assess one’s strengths and weaknesses and is an underlying feature of self-directed lifelong learning. However, the role of students’ self-evaluation remains controversial despite some attempts to clarify this aspect through comparisons with peer-review and faculty evaluations [1, 3, 7, 19]. Especially, the sex-based difference in self-assessment scores is still a controversial issue. Several studies revealed that students’ sex did not affect self-evaluation [10, 23–25]. However, other studies have found that gender does affect self-evaluation. Vivekananda-Schmidt et al. [26] and some other researchers advocated in their article that female students tend to underestimate themselves compared with male students on self-evaluation [12, 27]. Wiener et al. [15] and other studies [14, 28] reported that female students’ self-evaluations were higher than those of male students.

Dental schools in Japan adopt a 6-year course program comprising 12 semesters. Students enter dental school immediately after graduation from high school. A typical example of the dental school curriculum is as follows. The first year involves general sciences, with preclinical training in the second to fourth years, clinical practicum at the university clinic in the fifth year, and classroom lectures to prepare for the National Board examination in the sixth year. In the second semester of the fourth year at the Nippon Dental University School of Dentistry at Niigata, students complete a “communication skills in clinical dentistry” practical training course. This course consists of three parts: medical interviews (five units), cooperation among healthcare professionals (1 unit), and treatment plan report (1 unit). All students spend 3 h per week on each unit in the course.

This study aimed to investigate students’ potential for self-evaluation in a clinical dentistry practical training course on communication skills in a Japanese dental school. To our knowledge, no studies have examined Japanese dental students during a clinical dentistry practical training course on communication skills. Specifically, we analyzed possible differences between male and female students. The null hypotheses were as follows: there was no difference between male and female students’ self-evaluation scores and there was no difference between students’ self-evaluation and faculty evaluation.

## Materials and Methods

Approval for this study was obtained from the Research Ethics Committee of The Nippon Dental University, School of Life Dentistry at Niigata (Approval No. ECNG-E-3). Participants were fourth-year students in 2017 and 2018 at the Nippon Dental University, School of Life Dentistry at Niigata. Students in 2017 and 2018 were different cohorts, and a total number of students were 142 (84 males, 58 females; all Japanese). Students who stayed in the same class for another year (14 males, four females; all Japanese) were excluded. Finally, the 124 students (70 males, 54 females; all Japanese) who participated in this study signaled their agreement to participate by completing a consent form.

Table 1 shows the course syllabus for the communication skills in clinical dentistry practical training course. This three-unit course is held once per week, and students spend 3 h on each unit. Participating students were asked to complete a self-evaluation sheet at the end of each unit, which was at weekly intervals.

**Table 1:** Communication skills in clinical dentistry practical training course syllabus

Unit	Theme	Content	Method
1-1	Introduction to medical interviews	Medical interviews for narrative-based medicine	Role play and group discussion
1-2	Medical interview (chronic symptoms)	Interviewing a person with chronic symptoms to obtain their medical history	Role play and group discussion
1-3	Medical interview (acute symptoms)	Interviewing a person with acute symptoms to obtain their medical history	Role play and group discussion
2	Cooperation among healthcare professionals	Producing documents (referral forms, prescriptions, and technical material order forms)	Simulation and group discussion
3	Treatment plan report	Reporting a treatment plan for a prosthesis to a patient and obtaining informed consent	Role play and group discussion
1-4	Medical interview (examination)	Interviewing a simulated patient to obtain their medical history	Practical examination
1-5	General overview of medical interviews	Looking back over the examples of typical symptoms for a medical interview	Role play, simulation, and group discussion

On the first day of communication skills in clinical dentistry practical training course, students were informed about the evaluation items and their criterion using slides through a PowerPoint presentation and printed matter. The evaluation items and their criterion are shown in Table 2. The self-evaluation sheet includes a 4-point Likert scale (1 = poor, 2 = fair, 3 = good, 4 = excellent), and participating students were asked to complete the self-evaluation for each evaluation items on the 4-point Likert scale [6, 17]. Faculties evaluate students on the 4-point Likert scale with the same criterion of evaluation items. Through the “communication skills in clinical dentistry practical training course,” the same evaluation sheet and items were used for evaluation. At the end of each unit, faculty feedback to the students was made including self-evaluation scores. At the beginning of the next unit, face to face individual feedback was made, if there was a two-point difference in the score between student self-evaluation and faculty evaluation on the Likert scale.

**Table 2:** The criterion for evaluation

		Score 1 (poor)	Score 2 (fair)	Score 3 (good)	Score 4 (excellent)
Tidy clothes as a dental student		Less than standard	Almost meet the criteria but not enough	Clean and dignified clothes	Clean and dignified clothes as a dental student
Manner as a dental student		Very crude attitude	Crude attitude	Has manner as a preclinical dental student	Has excellent manner as a dental student
Role play	Independence	Frequently ask someone what to do	Sometimes depending on others but almost independent	Positively perticipate in the role play	Positively participate in the role play, with consideration for others
	Cooperativeness	Self-centered and self-absorbed	Somewhat self-centered and self-absorbed	Cooperate respectfully and amicably	Cooperate respectfully and amicably with an excellent manner
	Sincere attitude	frivolous attitude; chatting frequently, dozing, etc.	chatting sometimes, need more concentration	With good concentration	With excellent concentration
	Good communication	Not to communicate with others	Tried to communicate with others, but not enough	Communicate with others, respectfully and amicably	Communicate with others, respectfully and amicably as a student doctor
Small-group discussion	Participation	No willingness to make comments	Have the willingness to make comments, but not enough	Actively make comments and engaged in the discussion	Frequently make comments and actively engaged in the discussion
	Validity of statement	Statements have poor validation	Statements have evidence but somewhat precarious	Statements were valid and precise	Statements were accurate and beyond the score 3
	Attitude to hearing others’ opinions	Don’t feel like listening to others	Sometimes concentrate on the other opinions	Concentrate on the other opinions	Actively listen to the opinion of others
	Good communication	Not to communicate with others	Tried to communicate with others, but not enough	Communicate with others, respectfully and amicably	Communicate with others, respectfully and amicably as a student doctor
		Score 5 (poor)	Score 10 (fair)	Score 15 (good)	Score 20 (excellent)
Overall attitude		Poor behavior, overall	good attitude, somewhat	Good attitude overall as a preclinical dental student	Excellent attitude overall like a student doctor

Participating students were evaluated by six tutors (three males, three females). The same six tutors evaluated students in both 2017 and 2018. Regarding the students’ evaluation, each tutor was responsible for evaluating 20 to 22 students. Some of the evaluation items are subjective and the tutors evaluate students with the viewpoints of clinical dentists. The tutors have over 10 years of clinical experience as the clinical dentists and they were well trained in obtaining concordance of student evaluation. The six tutors confirmed the evaluation criterion prior to the start of the “communication skills in clinical dentistry practical course” in each year, with evaluating the simulated student acted by the other faculty member. They have over 10 years of experience of student evaluation in the “communication skills in clinical dentistry practical course.”

Self-evaluation scores and faculty evaluation scores for each student for Units 1-1, 1-2, and 1-3 were statistically analyzed. First, mean value comparisons for self-evaluation and faculty evaluation scores were calculated and compared between males and females. Second, we clarified the change over time in mean values for students’ self-evaluation and faculty evaluation scores. Finally, we compared the mean values of students’ self-evaluation scores and faculty evaluation scores.

### Statistical Analysis

Mann-Whitney *U* tests were used to compare the mean values for the self-evaluation and faculty evaluation scores between males and females. Friedman and Scheffe tests were used to clarify the change over time in the mean values of students’ self-evaluation and faculty evaluation scores. If a significant difference was observed, a Scheffe multiple comparison test was performed to identify where there were significant differences in each group. Wilcoxon signed-rank tests were used to compare the mean values for male and female students between students’ self-evaluation and faculty evaluation scores. The level of significance (alpha) was set at 0.05 for all statistical analyses.

The sample size was calculated based on a power analysis using G*Power software version 3.1.9.2 (Heinrich Heine University, Dusseldorf, Germany). The power analysis for a Wilcoxon signed-rank test at an alpha error probability of 0.05 (effect size = 0.5) showed that the actual power was 0.951 for female students and 0.980 for male students. The power analysis for the Mann-Whitney *U* test at an alpha error probability of 0.05 (effect size = 0.8) showed that the actual power was 0.980 for female students and 0.996 for male students. Finally, the power analysis for a Friedman test at an alpha error probability of 0.05 (effect size = 0.25) showed that the actual power was 0.923 for sex-based differences. These results demonstrated that the sample size in each group was sufficient for statistical comparisons [29]. All statistical analyses were performed using the software Bell Curve for Excel (Social Survey Research Information Co., Ltd., Tokyo, Japan).

## Results

There were no students with a two-point difference in the score between student self-evaluation and faculty evaluation on the Likert scale at the end of the unit so that no face to face individual feedback was made at the beginning of the next unit. Table 3 presents the sex-based differences in students’ self-evaluation. The self-evaluation scores were significantly higher in females than in males, with this phenomenon being especially pronounced in Unit 1-3. Table 4 shows the sex-based differences in faculty evaluations. For the evaluation items concerning tidy clothes and manner as a dental student, faculty evaluation scores for female students were higher than those for male students. For the evaluation items concerning independence in role play and overall attitude, scores for female students were higher than those for males in Units 1-1 and 1-2, whereas no significant differences were observed in Unit 1-3.

**Table 3:** Sex-based differences in students’ self-evaluation

Evaluation items	Unit 1-1					Unit 1-2					Unit 1-3				
	Male students			Female students		Male students			Female students		Male students			Female students	
	Mean	SD		Mean	SD	Mean	SD		Mean	SD	Mean	SD		Mean	SD
Tidy clothes as a dental student	3.91	0.28	NS	3.98	0.14	3.86	0.35	*p* < 0.05	3.96	0.19	3.83	0.38	*p* < 0.05	3.96	0.19
Manner as a dental student	3.83	0.38	*p* < 0.05	3.96	0.19	3.87	0.34	NS	3.94	0.23	3.81	0.39	*p* < 0.05	3.94	0.23
Role play															
Independence	3.77	0.46	NS	3.83	0.38	3.77	0.46	NS	3.81	0.39	3.67	0.47	*p* < 0.05	3.85	0.36
Cooperativeness	3.70	0.49	NS	3.83	0.42	3.83	0.38	NS	3.87	0.34	3.76	0.43	*p* < 0.05	3.89	0.37
Sincere attitude	3.67	0.47	NS	3.80	0.41	3.77	0.46	NS	3.85	0.36	3.73	0.45	*p* < 0.05	3.91	0.29
Good communication	3.69	0.55	NS	3.80	0.41	3.79	0.45	NS	3.89	0.32	3.71	0.46	*p* < 0.05	3.87	0.34
Small-group discussion															
Participation	3.69	0.53	NS	3.81	0.39	3.74	0.50	NS	3.83	0.38	3.63	0.52	NS	3.72	0.49
Validity of statement	3.64	0.48	NS	3.67	0.48	3.73	0.48	NS	3.65	0.48	3.67	0.47	NS	3.72	0.45
Attitude to hearing others’ opinions	3.79	0.41	NS	3.91	0.29	3.71	0.49	*p* < 0.01	3.94	0.23	3.77	0.42	*p* < 0.05	3.93	0.26
Good communication	3.74	0.47	NS	3.87	0.34	3.71	0.49	*p* < 0.05	3.91	0.29	3.69	0.50	NS	3.78	0.46
Overall attitude	16.43	4.83	NS	17.59	4.32	17.43	4.40	NS	17.78	4.20	16.86	4.68	NS	17.41	4.42

*SD*, standard deviation; *NS*, not significant

Number of students: 70 males, 54 females

**Table 4:** Sex-based differences in faculty evaluation

Evaluation items	Unit 1-1					Unit 1-2					Unit 1-3				
	Male students			Female students		Male students			Female students		Male students			Female students	
	Mean	SD		Mean	SD	Mean	SD		Mean	SD	Mean	SD		Mean	SD
Tidy clothes as a dental student	3.93	0.26	NS	3.98	0.14	3.69	0.50	NS	3.72	0.45	3.57	0.50	*p* < 0.01	3.78	0.50
Manner as a dental student	3.91	0.33	NS	3.98	0.14	3.51	0.56	NS	3.69	0.47	3.49	0.56	*p* < 0.01	3.80	0.41
Role play															
Independence	3.44	0.50	*p* < 0.05	3.65	0.48	3.15	0.50	*p* < 0.01	3.80	0.41	3.44	0.53	NS	3.44	0.54
Cooperativeness	3.61	0.55	NS	3.80	0.41	3.59	0.50	NS	3.74	0.44	3.50	0.53	NS	3.69	0.47
Sincere attitude	3.51	0.63	NS	3.69	0.47	3.46	0.56	NS	3.61	0.49	3.30	0.71	NS	3.44	0.66
Good communication	3.47	0.53	*p* < 0.05	3.28	0.45	3.59	0.50	NS	3.50	0.51	3.49	0.53	NS	3.52	0.54
Small-group discussion															
Participation	3.49	0.53	NS	3.59	0.50	3.66	0.51	NS	3.74	0.44	3.36	0.57	NS	3.33	0.58
Validity of statement	3.37	0.49	NS	3.44	0.50	3.33	0.56	*p* < 0.01	3.61	0.49	3.39	0.60	NS	3.43	0.57
Attitude to hearing others’ opinions	3.56	0.53	NS	3.63	0.49	3.57	0.53	NS	3.65	0.48	3.54	0.53	NS	3.63	0.49
Good communication	3.49	0.50	NS	3.50	0.51	3.53	0.50	*p* < 0.05	3.70	0.46	3.54	0.50	NS	3.44	0.50
Overall attitude	14.36	5.10	*p* < 0.01	17.04	4.61	14.50	5.12	*p* < 0.01	17.78	4.20	15.50	5.12	NS	16.67	4.76

*SD*, standard deviation; *NS*, not significant

Number of students: 70 males, 54 females

Number of faculties: 3 males, 3 females

The self-evaluation for male and female students is shown in Table 5. For female students, a significant difference was observed between Units 1-2 and 1-3 for the item good communication in small-group discussion. The faculty evaluation for students is shown in Table 6. For the evaluation items of tidy clothes and manner as a dental student, significant differences were observed between Units 1-1 and 1-2 between males and females. Significant differences were also observed between Units 1-2 and 1-3 between males and females for participation in small-group discussion. For independence in role play and good communication in small-group discussion, there were significant differences in female students between Units 1-2 and 1-3. For the evaluation item concerning good communication in role play, a significant difference was observed in female students between Units 1-1 and 1-3.

**Table 5:** Students’ self-evaluation

Evaluation items	Gender	Unit 1-1		Unit 1-2		Unit 1-3		Friedman test	Comparison between measurement times			
		Mean	SD	Mean	SD	Mean	SD		Scheffe	1-1 vs. 1-2	1-1 vs. 1-3	1-2 vs. 1-3
Tidy clothes as a dental student	Males	3.91	0.28	3.86	0.35	3.83	0.38	NS				
	Females	3.98	0.14	3.96	0.19	3.96	0.19	NS				
Manner as a dental student	Males	3.83	0.38	3.87	0.34	3.81	0.39	NS				
	Females	3.96	0.19	3.94	0.32	3.94	0.23	NS				
Role play												
Independence	Males	3.77	0.46	3.77	0.46	3.67	0.47	NS				
	Females	3.83	0.38	3.82	0.39	3.85	0.36	NS				
Cooperativeness	Males	3.70	0.49	3.83	0.38	3.76	0.43	NS				
	Females	3.83	0.42	3.87	0.34	3.89	0.37	NS				
Sincere attitude	Males	3.67	0.47	3.77	0.46	3.73	0.45	NS				
	Females	3.80	0.41	3.85	0.36	3.91	0.29	NS				
Good communication	Males	3.69	0.55	3.79	0.45	3.71	0.46	NS				
	Females	3.80	0.41	3.89	0.32	3.87	0.34	NS				
Small-group discussion												
Participation	Males	3.69	0.53	3.74	0.50	3.63	0.52	NS				
	Females	3.82	0.39	3.83	0.38	3.72	0.49	NS				
Validity of statement	Males	3.64	0.48	3.74	0.48	3.67	0.47	NS				
	Females	3.67	0.48	3.65	0.48	3.72	0.45	NS				
Attitude to hearing others’ opinions	Males	3.79	0.41	3.71	0.49	3.77	0.42	NS				
	Females	3.91	0.29	3.94	0.23	3.93	0.26	NS				
Good communication	Males	3.74	0.47	3.71	0.49	3.69	0.50	NS				
	Females	3.87	0.34	3.91	0.29	3.78	0.46	*p* < 0.05		NS	NS	*p* < 0.05
Overall attitude	Males	16.43	4.83	17.43	4.40	16.86	4.68	NS				
	Females	17.59	4.32	17.78	4.20	17.41	4.42	NS				

*SD*, standard deviation; *NS*, not significant

Number of students: 70 males, 54 females

**Table 6:** Faculty evaluation of students

Evaluation items	Gender	Unit 1-1		Unit 1-2		Unit 1-3		Friedman test	Comparison between measurement times			
		Mean	SD	Mean	SD	Mean	SD		Scheffe	1-1 vs. 1-2	1-1 vs. 1-3	1-2 vs. 1-3
Tidy clothes as a dental student	Male students	3.93	0.26	3.69	0.50	3.57	0.50	*p* < 0.01		*p* < 0.01	*p* < 0.01	NS
	Female students	3.98	0.14	3.72	0.45	3.78	0.50	*p* < 0.01		*p* < 0.01	*p* < 0.05	NS
Manner as a dental student	Male students	3.91	0.33	3.51	0.56	3.49	0.56	*p* < 0.01		*p* < 0.01	*p* < 0.01	NS
	Female students	3.98	0.14	3.69	0.47	3.80	0.41	*p* < 0.01		*p* < 0.01	*p* < 0.05	NS
Role play												
Independence	Male students	3.44	0.50	3.15	0.50	3.44	0.53	NS				
	Female students	3.65	0.48	3.80	0.41	3.44	0.54	*p* < 0.01		NS	NS	*p* < 0.01
Cooperativeness	Male students	3.61	0.55	3.59	0.50	3.50	0.53	NS				
	Female students	3.80	0.41	3.74	0.44	3.69	0.47	NS				
Sincere attitude	Male students	3.51	0.63	3.46	0.56	3.30	0.71	NS				
	Female students	3.69	0.47	3.61	0.49	3.44	0.66	NS				
Good communication	Male students	3.47	0.53	3.59	0.50	3.49	0.53	NS				
	Female students	3.28	0.45	3.50	0.51	3.52	0.54	*p* < 0.05		NS	*p* < 0.05	NS
Small-group discussion												
Participation	Male students	3.49	0.53	3.66	0.51	3.36	0.57	*p* < 0.01		NS	NS	*p* < 0.01
	Female students	3.59	0.50	3.74	0.44	3.33	0.58	*p* < 0.01		NS	NS	*p* < 0.01
Validity of statement	Male students	3.37	0.49	3.33	0.56	3.39	0.60	NS				
	Female students	3.44	0.50	3.61	0.49	3.43	0.57	NS				
Attitude to hearing others’ opinions	Male students	3.56	0.53	3.57	0.53	3.54	0.53	NS				
	Female students	3.63	0.49	3.65	0.48	3.63	0.49	NS				
Good communication	Male students	3.49	0.50	3.53	0.50	3.54	0.50	NS				
	Female students	3.50	0.51	3.70	0.46	3.44	0.50	*p* < 0.05		NS	NS	*p* < 0.05
Overall attitude	Male students	14.36	5.10	14.50	5.12	15.50	5.12	NS				
	Female students	17.04	4.61	17.78	4.20	16.67	4.76	NS				

*SD*, standard deviation; *NS*, not significant

Number of students: 70 males, 54 females

Number of faculties: 3 males, 3 females

Table 7 presents the differences between male students’ self-evaluation and faculty evaluations. The evaluation items with significant differences between male students’ self-evaluation and faculty evaluation increased from Unit 1-1 to Unit 1-3. For Unit 1-3, significant differences were observed in nine of the 11 evaluation items. Table 8 shows the differences between female students’ self-evaluation and faculty evaluations. The evaluation items with a significant difference between female students’ self-evaluation and faculty evaluations increased from Unit 1-1 to Unit 1-3. For Unit 1-3, significant differences were observed for 10 of the 11 evaluation items.

**Table 7:** Male students’ self-evaluation and faculty evaluation

Evaluation items	Unit 1-1					Unit 1-2					Unit 1-3				
	Male students			Faculty		Male students			Faculty		Male students			Faculty	
	Mean	SD		Mean	SD	Mean	SD		Mean	SD	Mean	SD		Mean	SD
Tidy clothes as a dental student	3.91	0.28	NS	3.93	0.26	3.86	0.35	*p* < 0.05	3.69	0.50	3.83	0.38	*p* < 0.01	3.57	0.50
Manner as a dental student	3.83	0.38	NS	3.91	0.33	3.87	0.34	*p* < 0.01	3.51	0.56	3.81	0.39	*p* < 0.01	3.49	0.56
Role play															
Independence	3.77	0.46	*p* < 0.01	3.44	0.50	3.77	0.46	*p* < 0.01	3.15	0.50	3.67	0.47	*p* < 0.01	3.44	0.53
Cooperativeness	3.70	0.49	NS	3.61	0.55	3.83	0.38	*p* < 0.01	3.59	0.50	3.76	0.43	*p* < 0.01	3.50	0.53
Sincere attitude	3.67	0.47	NS	3.51	0.63	3.77	0.46	*p* < 0.01	3.46	0.56	3.73	0.45	*p* < 0.01	3.30	0.71
Good communication	3.69	0.55	NS	3.47	0.53	3.79	0.45	*p* < 0.05	3.59	0.50	3.71	0.46	*p* < 0.05	3.49	0.53
Small-group discussion															
Participation	3.69	0.53	*p* < 0.05	3.49	0.53	3.74	0.50	NS	3.66	0.51	3.63	0.52	*p* < 0.01	3.36	0.57
Validity of statement	3.64	0.48	*p* < 0.01	3.37	0.49	3.73	0.48	*p* < 0.01	3.33	0.56	3.67	0.47	*p* < 0.05	3.39	0.60
Attitude to hearing others’ opinions	3.79	0.41	*p* < 0.01	3.56	0.53	3.71	0.49	NS	3.57	0.53	3.77	0.42	*p* < 0.01	3.54	0.53
Good communication	3.74	0.47	*p* < 0.01	3.49	0.50	3.71	0.49	NS	3.53	0.50	3.69	0.50	NS	3.54	0.50
Overall attitude	16.43	4.83	*p* < 0.05	14.36	5.10	17.43	4.40	*p* < 0.01	14.50	5.12	16.86	4.68	NS	15.50	5.12

*SD*, standard deviation; *NS*, not significant

Number of students: 70 males, 54 females

Number of faculties: 3 males, 3 females

**Table 8:** Female students’ self-evaluation and faculty evaluation

Evaluation items	Unit 1-1					Unit 1-2					Unit 1-3				
	Female students			Faculty		Female students			Faculty		Female students			Faculty	
	Mean	SD		Mean	SD	Mean	SD		Mean	SD	Mean	SD		Mean	SD
Tidy clothes as a dental student	3.98	0.14	NS	3.98	0.14	3.96	0.19	*p* < 0.01	3.72	0.45	3.96	0.19	*p* < 0.05	3.78	0.50
Manner as a dental student	3.96	0.19	NS	3.98	0.14	3.94	0.23	*p* < 0.01	3.69	0.47	3.94	0.23	*p* < 0.05	3.80	0.41
Role play															
Independence	3.83	0.38	*p* < 0.05	3.65	0.48	3.81	0.39	NS	3.80	0.41	3.85	0.36	*p* < 0.01	3.44	0.54
Cooperativeness	3.83	0.42	NS	3.80	0.41	3.87	0.34	NS	3.74	0.44	3.89	0.37	*p* < 0.05	3.69	0.47
Sincere attitude	3.80	0.41	NS	3.69	0.47	3.85	0.36	*p* < 0.05	3.61	0.49	3.91	0.29	*p* < 0.01	3.44	0.66
Good communication	3.80	0.41	*p* < 0.01	3.28	0.45	3.89	0.32	*p* < 0.01	3.50	0.51	3.87	0.34	*p* < 0.01	3.52	0.54
Small-group discussion															
Participation	3.81	0.39	*p* < 0.05	3.59	0.50	3.83	0.38	NS	3.74	0.44	3.72	0.49	*p* < 0.01	3.33	0.58
Validity of statement	3.67	0.48	*p* < 0.05	3.44	0.50	3.65	0.48	NS	3.61	0.49	3.72	0.45	*p* < 0.01	3.43	0.57
Attitude to hearing others’ opinions	3.91	0.29	*p* < 0.01	3.63	0.49	3.94	0.23	*p* < 0.01	3.65	0.48	3.93	0.26	*p* < 0.01	3.63	0.49
Good communication	3.87	0.34	*p* < 0.01	3.50	0.51	3.91	0.29	*p* < 0.05	3.70	0.46	3.78	0.46	*p* < 0.01	3.44	0.50
Overall attitude	17.59	4.32	NS	17.04	4.61	17.78	4.20	NS	17.78	4.20	17.41	4.42	NS	16.67	4.76

*SD*, standard deviation; *NS*, not significant

Number of students: 70 males, 54 females

Number of faculties: 3 males, 3 females

## Discussion

In this study, female students tended to rate significantly higher self-assessment scores than male students. This tendency became stronger as the practical training progressed. Wiener et al. [15] also reported that female students’ self-evaluations were higher than those of male students. Our result was consistent with the reports that analyzed dental students [15] and medical students [14] in the USA. Our result was also consistent with the other study that analyzed non-medical students in Europe [28]. Our findings and these consistencies indicated that sex-based difference in the student’s self-evaluation is not affected by health promotion educational programs with different education systems and curricula. Colbert-Getz et al. [11] suggested that these scores were influenced by anxiety and confidence. Female students may perform better relative to male students because female students study more and are more prepared [30]. This suggests that female students in this study had more self-confidence and therefore tended to rate significantly higher self-assessment scores than male students. Female students may do better with several interpersonal aspects of medical care as Gruppen et al. reported [14]. On the other hand, Vivekananda-Schmidt et al. [26] and some other researchers advocated in their article that female students tend to underestimate themselves compared with male students on self-evaluation [12, 27]. Further research would be needed to clarify the relationship between sex and self-evaluation scores. Japanese dental students tended to prefer passive learning because of their self-restraint and reticence [31]. Traditionally, dentistry in Japan was a male-dominated profession and the percentage of female dental students was below 50%. In recent years, the number of female students is increasing in Japanese dental schools. Regarding the cultural and generational difference in gender roles, it may be that the learning preference and behavior of Japanese dental students may change as women assume a more assertive role in dentistry in Japan [32].

The results of the present study showed there were significant differences in evaluation scores between students and faculty in Unit 1-3 for nine of the 11 evaluation items for male students and 10 of the 11 items for female students. The results also showed that faculty evaluation scores decreased from Unit 1-1 to Unit 1-3. Emam et al. [17] suggested that overestimation in student’s self-evaluation may be attributed to those students having assessed their performance based on their memory and self-confidence that exceeded their expectations. The level of faculty expectations became higher from Unit 1-1 to Unit 1-3; however, students were satisfied with their performance based on their sense of achievement. Differences between student self-evaluation and faculty evaluation might be attributed to the students’ lesser understanding of the criteria used and not to the performance as such. In the present study, we employed just an evaluation sheet and a written criterion for evaluation, and we did not employ the rubric. There were no students with a two-point difference in the score between student self-evaluation and faculty evaluation on the Likert scale at the end of the unit so that no face to face individual feedback was made at the beginning of the next unit. Thus, it may be considered that the students were developing the assessment skills with group feedback and faculty feedback on the validity of their assessment. The rubric might support student’s self-evaluation as examples, to explain the various levels of attainment. The rubric implies both student and faculty what is important and thereby give clarity and explicitness to the assessment [33–35]. It may be considered that the use of rubric has the potential of promoting learning and/or improving instruction, at least as perceived by the students and faculties using them. How rubrics support learning and instruction is by making expectations and criteria explicit, which also facilitates feedback and self-evaluation [36]. In further research after employing the rubric in the “communication skills in clinical dentistry practical course,” we would like to ascertain the effect of that to students’ self-evaluation and faculty evaluation.

Based on Bandura’s theory [37], Hadid [10] reported that self-efficacy affects self-evaluation, which implies that students who believe they can perform a certain task usually do not experience negative thoughts about their ability to perform that task successfully. Self-evaluation-based self-assessment is an important skill for dentists in daily clinical practice to provide effective oral care [38]. The ability to accomplish self-assessment requires training and practice and helps the professional to understand their strengths and weaknesses [39]. The validity of self-assessment is important for health professionals looking to self-directed life-long learning as a source of continuing professional vitality [40].

The present study was limited in that the two cohorts were restricted to students at one dental university. Although this study was conducted over two academic years, our results represent a small sample of students. Due to the limitation of the sample size of involved students and numbers of faculty who participate in the “communication skills in clinical dentistry practical course,” this study was not able to ascertain whether the gender of the faculty member influenced the assessment of students of the other gender. In this study, the faculty evaluation of the students was employed, not the control group of the students. McKenzie et al. [18] set the intervention group and no intervention/control group in their research. In their study, the intervention group was a class of 2016 and no intervention/control group was a class of another year (2015). On the other hand, the research papers without control group settings were published [2, 10, 15, 17, 35]. Further study would be needed for the comparison of students’ self-evaluation skills between the intervention group and no intervention/control group to analyze the Japanese students’ behavior. So, we analyzed the student classes of 2017 and 2018 in the present study for the control group of further research. Wagner et al. [7] noted that the self-assessment process should be validated; students should receive adequate training on how to use these skills and be enabled to practice self-assessment frequently. Additionally, students’ self-assessment skills were still developing because they were fourth-year students in a 6-year dental school curriculum. All of the tutors who participated in the “communication skills in clinical dentistry practical training course” belonged to the clinical department of our university, and they treat patients in the university hospital. Thus, it is possible that the tutors can check later year students and young dentists exhibit the intended behaviors or not. During the internship of dental students, it may be considered that the students’ behaviors reinforced in their clinical experiences. In further research, we would like to clarify this point as well. Despite these limitations, the results of this study indicated that students’ self-assessment offered the possibility of enhancing their ability for self-directed dentistry learning, and also offered insight as to how faculty can effectively educate dental students. Although further studies are required, we believe that dental educators need to assume the responsibility of helping students to develop skills in decision-making, communication, professionalism, and reflection for lifelong learning [20].

The null hypothesis of the present study that there was no difference between male and female students’ self-evaluation scores was rejected. Also, the null hypothesis that there was no difference between student’s self-evaluation and faculty evaluation was rejected. Dental educators should support students to increase their levels of self-efficacy to enhance their self-evaluation skills. Enhancing students’ self-evaluation skills may lead to improvements in student’s learning skills and learning approaches for better academic performance.
